# Multitasking during social interactions in adolescence and early adulthood

**DOI:** 10.1098/rsos.150117

**Published:** 2015-11-25

**Authors:** Kathryn L. Mills, Iroise Dumontheil, Maarten Speekenbrink, Sarah-Jayne Blakemore

**Affiliations:** 1 Institute of Cognitive Neuroscience, University College London, London, UK; 2 Experimental Psychology, University College London, London, UK; 3 Department of Psychological Sciences, Birkbeck College, University of London, London, UK; 4 Child Psychiatry Branch, National Institute of Mental Health, Bethesda, MD, USA

**Keywords:** cognitive load, development, dual task, egocentric bias, working memory

## Abstract

Multitasking is part of the everyday lives of both adolescents and adults. We often multitask during social interactions by simultaneously keeping track of other non-social information. Here, we examined how keeping track of non-social information impacts the ability to navigate social interactions in adolescents and adults. Participants aged 11–17 and 22–30 years old were instructed to carry out two tasks, one social and one non-social, within each trial. The social task involved referential communication, requiring participants to use social cues to guide their decisions, which sometimes required taking a different perspective. The non-social task manipulated cognitive load by requiring participants to remember non-social information in the form of one two-digit number (low load) or three two-digit numbers (high load) presented before each social task stimulus. Participants showed performance deficits when under high cognitive load and when the social task involved taking a different perspective, and individual differences in both trait perspective taking and working memory capacity predicted performance. Overall, adolescents were less adept at multitasking than adults when under high cognitive load. These results suggest that multitasking during social interactions incurs performance deficits, and that adolescents are more sensitive than adults to the effects of cognitive load while multitasking.

## 1. Introduction

The ability to navigate social interactions continues to develop throughout human adolescence [1]. For both adolescents and adults, many social interactions involve multitasking, such as keeping track of extraneous information while engaging in a conversation or remembering a phone number while taking directions from someone. Social interactions involve attending to social cues and often require taking a perspective that differs from one’s own. Previous work has shown that the tendency to take a different perspective in social interactions continues to increase during adolescence and into young adulthood [2]. However, it is unknown whether the ability to process social cues or take a different perspective is affected by multitasking during social situations, and if this relationship changes across development. In this study, we examined how keeping track of non-social information affects the ability to attend to social cues and adopt another person’s perspective during social interactions in a group of adolescents and young adults.

While attending to social cues is largely automatic [3], taking another person’s perspective when it differs from one’s own requires inhibiting our own, egocentric, perspective [4]. For example, telling a story to a friend requires keeping track of any background information that she might not know. As humans are susceptible to *epistemic egocentrism* [5], inhibiting an egocentric perspective is effortful and requires cognitive resources for executive control, such as working memory (WM) [6]. We can measure the availability of cognitive resources in an individual by assessing their WM capacity. We can also experimentally manipulate the availability of cognitive resources in an individual by asking them to simultaneously perform a task that taxes their WM capacity (cognitive load). Previous studies have shown that adults with higher WM capacity are better at inhibiting their egocentric perspective in live social interactions than individuals with lower WM capacity, and that this ability is disrupted in both groups when placed under high cognitive load [7]. These results suggest that multitasking during a social interaction task would impair performance only when cognitive resources are sufficiently taxed. As both the tendency to take someone else’s perspective [2] and the ability to manipulate information in WM [8,9] are still developing in adolescence, we hypothesized that adolescents would be more affected by cognitive load than adults when keeping track of others’ perspectives during social interactions.

**Figure 1.**
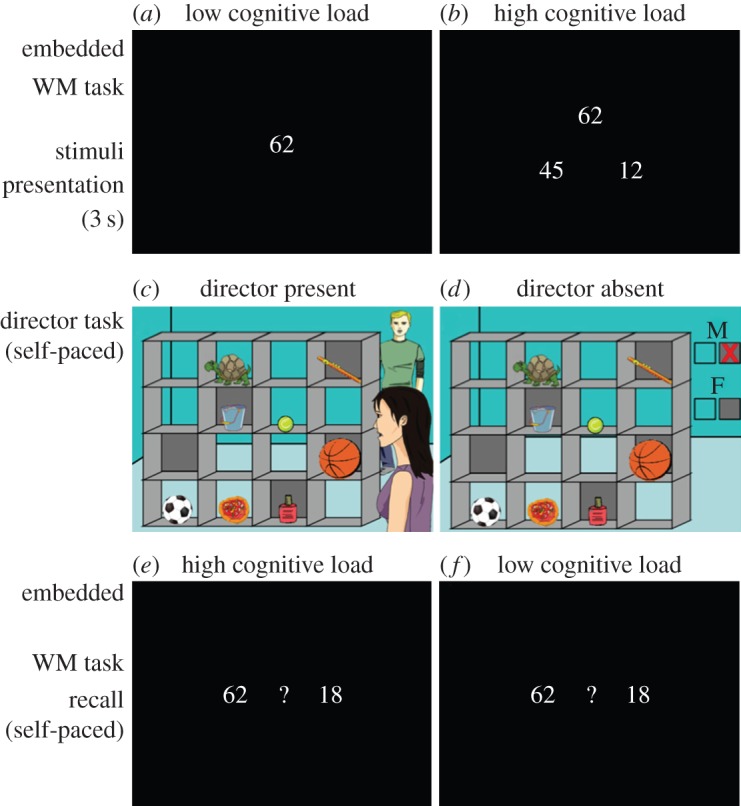
Presentation of multitasking paradigm. For each trial, participants were first presented with either (*a*) one two-digit number (low load) or (*b*) three two-digit numbers (high load) for 3 s. Then participants were presented with the Director Task stimuli, which included a social (*c*) and non-social control condition (*d*). In this example, participants hear the instruction: ‘Move the large ball up’ in either a male or a female voice. If the voice is female, the correct object to move is the basketball, because in the DP condition the female director is standing in front of the shelves and can see all the objects, and in the DA condition, the absence of a red X on the grey box below the ‘F’ indicate that all objects can be moved by the participant. If the voice is male, the correct object to move is the football, because in the DP condition the male director is standing behind the shelves and therefore cannot see the larger basketball in the covered slot, and in the DA condition the red X over the grey box below the ‘M’ indicates that no objects in front of a grey background can be moved. After selecting an object in the Director Task, participants were presented with a display of two numbers, one of which corresponding to the only number (*e*) or one of the three numbers (*f*), shown to them at the beginning of the trial. Participants were instructed to click on the number they remembered being shown at the beginning of the trial.

In this study, we examined how the availability of cognitive resources affects multitasking during social interactions in adolescents and adults. Our multitasking procedure required participants to carry out two tasks (one social and one non-social) simultaneously. Our social task, called the Director Task (figure 1), required participants to interpret instructions from an avatar (called the ‘director’) to decide which object to move in a shelf array [10]. On half of the trials, the director’s perspective was different from that of the participant, and participants needed to take into account the director’s different perspective when deciding which object to move. The non-social task was a visual WM task in which two-digit numbers were visually presented before each Director Task stimulus. This non-social WM task also allowed us to manipulate the level of cognitive load by requiring participants to remember either one two-digit number (low load) or three two-digit numbers (high load). At the end of each trial, we assessed the participants’ ability to keep the number(s) in mind during the Director Task in order to obtain a measure of how successful participants were at multitasking during the social interaction. A matched rule-based variant of the Director Task that did not use social cues or necessitate perspective taking was used to differentiate between a general impact of cognitive control demands on performance from effects that specifically impact the social components of the task, i.e. using social cues and taking someone else’s perspective into account.

We also measured how natural variation in cognitive resources (assessed by WM capacity) related to multitasking performance. Participants completed a verbal reverse digit-span task as a measure of WM capacity (WM score) to assess individual differences in baseline cognitive resources. Finally, as the tendency to take a different perspective in social interactions continues to increase during adolescence and into young adulthood [2], we assessed how individual differences in trait perspective taking (TPT) related to multitasking performance.

## 2. Material and methods

### 2.1 Participants

We tested 30 female adults and 37 female adolescents. We excluded any outliers (determined by interquartile range) in individual traits: one adolescent was excluded because of low IQ and one adolescent because of low TPT. Two additional adults and two additional adolescents were excluded for performing lower than chance (33% accuracy) on at least one task condition. The entire dataset before exclusion criteria were applied and the analysis script detailing the exclusion procedure are available online (see Data accessibility). Data from 33 adolescents (11–17 years, mean age: 14.5±1.7) and 28 adults (22–30 years, mean age: 25.2±2.3) were included in the analysis (table 1). Participant groups did not differ in non-verbal IQ, measured by the matrix reasoning subtest of the Wechsler Abbreviated Scale of Intelligence (WASI) [11]. Adult participants were recruited through the UCL Psychology subject pool and adolescents through local advertisements.

**Table 1.** Demographic characteristics for adolescent and adult participant groups (all female).

	adolescents	adults
number of participants	33	28
age range (years)	11.3–17.8	22.4–30.0
mean age (years)	14.5 ± 1.7	25.2 ± 2.3
non-verbal IQ	108.6 ± 8.9	108.3 ± 8.7
WM score^a^	8.7 ± 3.7	11.6 ± 5.1
TPT^a^	15.5 ± 4.4	18.8 ± 3.6

^a^Significant differences between adolescent and adult groups at *p*<0.05.

### 2.2 Procedure

Participants completed four tasks in one laboratory visit. First, participants completed an adapted version of the Director Task (from [12]; figure 1) with an embedded WM Task component. Afterwards, participants completed a verbal reverse digit-span task to obtain a measure of trait verbal WM capacity (WM score), and the Interpersonal Reactivity Index (IRI) questionnaire to obtain a measure of TPT [13], and finally the matrix reasoning subtest of the WASI [11].

### 2.3 Director task with embedded working memory task

The Director Task with embedded WM Task followed a 2×2×2 factorial design, with three within-subject factors: Cognitive Load (low or high), Condition (director present (DP) or director absent (DA)) and Perspective (same or different).

Each trial started with presentation of the WM Task stimulus: either one two-digit number (low cognitive load; figure 1*a*) or three two-digit numbers (high cognitive load; figure 1*b*) were shown for 3 s.

Following the number display, participants were presented with the Director Task stimuli (48 total stimuli). These stimuli consisted of sets of 4×4 shelves with objects located in half of the slots. Five of the shelves had a grey background. On each trial, participants were given an instruction, by either a female or a male voice, to move one of the eight objects to a different slot in the shelves. The instructions referred to an object that was one of three exemplars in the shelves (e.g. the ball). In the DP condition, the display included two directors: one female and one male. One of them stood behind the shelves, facing the participant, whereas the other stood on the same side of the shelves as the participant (figure 1*c*). Out of the three exemplars of the object, one was always located in a slot with a grey background, not visible to the director standing behind the shelves. Consequently, only one of two objects could correspond to the heard instruction (‘Move the large ball up’), depending on the director’s viewpoint: an object in the closed slot if the director stood in front of the shelves, or an object in the clear slot if the director stood behind the shelves. Participants were instructed to use the position of the speaking director in order to determine which object to select and move. On half of the trials, the perspective of the director was the same as that of the participants (i.e. the director was standing in front of the shelves), on the other half the perspective of the director was different (i.e. the director was standing behind the shelves). Therefore, in the DP condition, participants had to consider the director’s position and perspective to determine the correct object to move.

In the DA condition, the auditory stimuli, object arrays and general instructions were the same as in the DP condition, but instead of directors alongside the shelves, there were two letters (‘M’ and ‘F’) with two boxes underneath each letter (figure 1*d*). These boxes served to indicate which boxes the participants could move objects from, depending on whether the male (‘M’) or the female (‘F’) gave the instruction. For example, the display in figure 1*d* informs participants that if the male is speaking they cannot move objects located in slots with a grey background (because the grey box is crossed out), while if the female is speaking they can move objects located in clear slots and those in slots with a grey background. These rules had precisely the same consequences as the position of the director in the DP blocks. Although perspective taking was not involved in the DA condition, for brevity we describe the manipulation of which object was the correct one to move (e.g. the largest of the three balls or the second largest visible to the director standing at the back/not in a slot with a grey background slot) as ‘perspective’ across DP and DA conditions for the analysis and results.

To vary the social and/or executive demands of the task, half of the Director Task trials included only one possibly correct object. In these trials, participants were shown arrays in which a unique target object was displayed in an open slot. In these trials, the director’s perspective in the DP condition, or the position of the X in the DA condition, made no difference to the correct interpretation of the instructions, and thus, participants could use their own perspective to select the appropriate object on all trials.

Each participant performed 16 blocks of 12 trials for a total of 192 trials. The blocks were mixed and counterbalanced for the DP and DA conditions. The same displays were used for the DP and DA conditions. The position of the directors (or the crossed box in the DA condition) was constant within blocks. However, cognitive load (low or high), the number of possibly target objects (1 or 3) and the gender of the speaker (female or male) varied trial by trial.

For each Director Task trial, the auditory instructions were presented with the visual stimulus over a period of 2.2 s, after which the visual stimulus remained on the screen until the participant made a selection—this was self-paced. Participants were instructed to listen to the instructions, select the correct object and then click-and-drag that object to the correct slot. Reaction times (RTs) were calculated as the delay between the presentation of the visual stimulus and the pressing of the mouse button. Accuracy only referred to which object was selected, and not whether it was moved to the correct slot, as the object selection is the measure of interest in this task.

After participants completed the Director Task portion of the trial, they were presented with a display of two numbers, one of which corresponded to the only number (low cognitive load; figure 1*e*), or one of the three numbers (high cognitive load; figure 1*f*), shown to them prior to the Director Task trial. Participants were instructed to click on the number they remembered being shown to them at the beginning of the trial.

### 2.4 Backward verbal digit-span task

Participants were instructed to repeat back sequences of three to seven numbers read aloud by the experimenter in reverse order. Numbers for the sequences were read aloud by the experimenter at 1 s intervals. After a practice trial with a sequence of two numbers, the sequences of numbers progressed by sets of four between three-span to six-span, whereas there were only two seven-span sequences. Participants had to remember two of the four sequences within each set to move onto the next set, or else the task was terminated. If a participant wrongly remembered the last sequence of one set and the first sequence of the next set, the task was terminated. We calculated each participant’s WM score as the number of correctly recalled sequences.

### 2.5 Interpersonal reactivity index questionnaire

The IRI consists of 28 self-reflective questions using 5-point scales, divided into four subscales [13]. For this study, we only used the score obtained on the Perspective Taking subscale. This subscale included questions such as: ‘I sometimes try to understand my friends better by imagining how things look from their perspective’ or ‘I sometimes find it difficult to see things from the ‘other guy’s’ point of view’.

### 2.6 Data analysis

We used mixed effects modelling to determine what factors best predicted multitasking performance. Accuracy was determined on a trial-by-trial basis, where a trial was considered accurate only if participants correctly performed both the Director Task and embedded WM Task. As our main interest was performance during social interactions, and not recall of non-social information, we analysed Director Task RT (correct trials only).

We used the lme4 package in R [14] to perform a linear mixed effects analysis on the relationship between our factors of interest and multitasking performance (accuracy and RT). Our factors of interest included three within-subject factors from the task: cognitive load (low versus high), condition (DA versus DP), perspective (same versus different); two individual traits: WM score and TPT; and one between-subjects factor: group (adolescents versus adults). As we hypothesized an interaction between WM score and TPT would relate to our task, we included a combined measure of these two individual traits by calculating and summing the ratios of TPT and the WM score (Combined Traits). We determined which factors best predicted performance for our measures of interest by testing global models including our factors of interest as fixed effects. Each model included a random intercept for each participant. Because of computational limitations, we performed a two-step procedure that involved five global models (table 2). First, all possible combinations of the variables within each of the five global models were tested using an automated model selection procedure (MuMIn1.9.0) [15]. Models were ranked using the Second-order Akaike Information Criterion (AICc) [16]. Second, the best-fitting models for each of the five global models were compared and ranked using AIC and likelihood ratio tests. All *p*-values reported in the main text were obtained by likelihood ratio tests comparing the best-fitting model against a baseline model that includes only the random effects and not the fixed effects of interest (see Data accessibility).

**Table 2.** Five Global Models. The five global models tested for each outcome of interest. When each of these models went through the automated search procedure, the potential main and interactive effects of each variable were compared. For example, the global search procedure for Global Model 1 compared the potential main and interactive effects for Group, Cognitive Load, Condition and Perspective. The difference between Model 4 and Model 5 is that Model 5 does not allow for individual main effects for TPT and WM Score, but instead only looks at the potential effects of an interaction between these two traits (Combined Traits). Asterisk (*) indicates main and interactive effects were explored between variables. Plus symbol (+) indicates only main effects were explored between variables.

global models
1. Group * Cognitive Load * Condition * Perspective
2. Group * Cognitive Load * Condition * Perspective + WM Score
3. Group * Cognitive Load * Condition * Perspective + TPT
4. Group * Cognitive Load * Condition * Perspective + WM Score + TPT
5. Group * Cognitive Load * Condition * Perspective + Combined Traits

### 2.7 Excluded trials

All errors were analysed and categorized. Trials in which errors were not specific to experimental instructions (e.g. the participant clicked outside of the shelf array) were excluded from the analysis (139 trials). Of the 48 stimuli, two were excluded because of deviant errors rates. We excluded trials in which participants responded faster than 600 ms (three trials), or slower than 20 s (three trials) during the Director Task. As trials with unique target objects were only included in this study to provide variety in the task instructions, we did not include these trials in our analyses. We analysed a total of 5539 trials.

## 3. Results

### 3.1 Individual traits

Mean scores on the Backward Verbal Digit-Span Task (WM score) and Perspective Taking subscale of the IRI (TPT) are reported in table 1. Overall, adolescent participants had lower WM scores than adults (*p*<0.01). Adolescent participants also reported having significantly lower TPT than adults (*p*<0.01).

### 3.2 Accuracy

**Figure 2.**
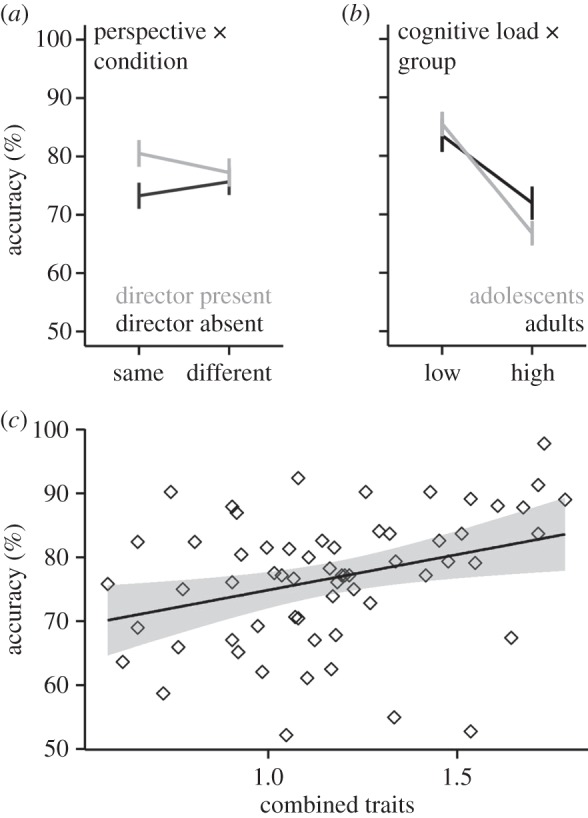
Multitasking accuracy results. (*a*) On average, there was an interaction between condition and perspective, driven by marginally (*p*=0.06) lower accuracy when using social cues (DP condition) to guide decisions when the perspective was different from their own, but no significant difference in accuracy (*p*=0.15) when using non-social cues (DA condition) to guide decisions. Regardless of cognitive load, participants were more accurate when using social cues to select the correct object compared to non-social cues (*p*<0.001), but only when participants shared the same perspective as the director. (*b*) On average, the adolescents’ multitasking accuracy was more affected by cognitive load than was the adults’. (*c*) The combined traits measure of WM score and TPT (*x*-axis shows summed ratios) was correlated with overall multitasking accuracy (*r*=0.33, *p*<0.01). Error bars represent 95% CIs.

Details of the best-fitting models for accuracy and RT are included in table 3. The best-fitting model for multitasking accuracy ($\chi_{7}^{2}=235$, *p*<0.001) included the main effects of group (adolescents versus adults), cognitive load (low versus high), condition (DA versus DP), perspective (same or different from the participant’s perspective) and the combined traits of WM score and TPT, as well as interactions between condition and perspective (figure 2*a*), and between group and cognitive load (figure 2*b* and table 3). On average, participants were less accurate when under high cognitive load, and when using non-social cues (DA condition) to guide decisions. The interaction between group and cognitive load revealed that, although both age groups showed poorer accuracy under high cognitive load (*ps*<0.001), adolescents’ multitasking accuracy was more affected by cognitive load than was adults’ (figure 2*b*). Across age groups, there was an interaction between condition and perspective (figure 2*a*). In the DP condition, participants were marginally less accurate when the perspective was different from their own compared to when it was the same (*p*=0.06), while perspective had no effect on accuracy in the DA condition (*p*=0.15), i.e. when using non-social cues to guide decisions. Following the condition by perspective interaction by comparing DP and DA conditions in same or different perspective trials instead showed that participants were more accurate when using social cues to select the correct object compared to non-social cues (*p*<0.001), but only when participants shared the same perspective as the director. There was no significant difference in accuracy (*p*=0.33) between DP and DA conditions when participants had to inhibit their own egocentric perspective to select the correct object. The combined traits measure of WM score and TPT was correlated with overall multitasking accuracy, in that a combination of higher WM score and higher TPT was associated with higher multitasking accuracy overall (*r*=0.33, *p*<0.01; figure 2*c*).

**Table 3.** Best-fitting models for multitasking accuracy and Director Task RT from correct multitasking trials only. The table displays the linear mixed model parameter estimates and standard errors (s.e.) of each fixed effect included in the best-fitting model. Model parameters reflect the influence of the following: Group: effect of adults completing the task compared to adolescents (baseline); Cognitive Load: effect of high cognitive load compared to low cognitive load (baseline); Condition: effect of DP compared to DA (baseline); Perspective: effect of different perspective compared to same perspective (baseline); Combined Traits: the summed ratios of TPT and WM score. Significant fixed effects are highlighted in bold. *Z*-values are reported for binomial models (accuracy) and *t*-values are reported for linear models (RT).

	accuracy			
fixed effects	estimate	s.e.	*z*-value	*p*-value
intercept	0.80	0.32	2.49	**0**.**013**
group (adults versus adolescents)	−0.33	0.19	−1.71	0.087
cognitive load (high versus low)	−1.12	0.09	–11.92	**<0**.**001**
condition (DP versus DA)	0.42	0.10	4.36	**<0**.**001**
perspective (different versus same)	0.11	0.09	1.18	0.237
combined traits	0.83	0.28	2.93	**0**.**003**
group × cognitive load	0.39	0.14	2.83	**0**.**005**
condition × perspective	−0.27	0.13	−2.01	**0**.**045**

	Director Task RT (correct trials only)			
fixed effects	estimate	s.e.	*t*-value	*p*-value
intercept	3844.80	83.70	45.94	**<0**.**001**
cognitive load (high versus low)	138.10	66.30	2.08	**0**.**037**
condition (DP versus DA)	−305.90	61.40	−4.99	**<0**.**001**
perspective (different versus same)	576.30	60.70	9.50	**<0**.**001**
cognitive load × condition	−275.70	91.40	−3.02	**0**.**003**
cognitive load × perspective	35.30	91.60	0.39	0.700
condition × perspective	−33.80	85.90	−0.39	0.694
cognitive load × condition × perspective	287.60	127.20	2.26	**0**.**024**

### 3.3 Reaction time

**Figure 3.**
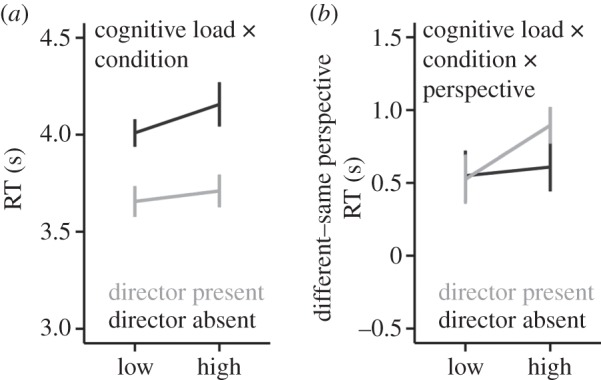
Director Task RT results. We analysed RT data from the Director Task for correct multitasking trials. (*a*) In both the low cognitive load and high cognitive load conditions, participants were faster at the Director Task when using social cues to select the correct object than when using the non-social cues (*p*<0.001). When using non-social cues (DA condition) to guide decisions, participants were slower under high cognitive load compared with low cognitive load (*p*=0.03), whereas cognitive load did not seem to affect RT for trials in which participants had to use social cues (DP condition) to guide decisions (*p*=0.30). (*b*) When under high cognitive load, participants were slower at selecting the correct object when taking a different perspective compared with when they did not have to take a different perspective (*p*<0.001), and this effect was present only when using social cues to select the correct object.

We analysed RT data from the Director Task for correct multitasking trials. The best-fitting model for RT during correct multitasking trials ($\chi_{7}^{2}=561$, *p*<0.001) included the main effects of cognitive load, condition and perspective, as well as a two-way interaction between cognitive load and condition, and a three-way interaction between cognitive load, condition and perspective (figure 3 and table 3). On average, participants were slower under high cognitive load, when using non-social cues (DA condition) to guide decisions, and when they had to adopt a different perspective. Following up the cognitive load × condition interaction indicated that, in both the low cognitive load and high cognitive load conditions, participants were faster at the Director Task when using social cues to select the correct object than when using the non-social cues (*ps*<0.001). When using non-social cues (DA condition) to guide decisions, participants were slower under high cognitive load compared with low cognitive load (*p*=0.03), whereas cognitive load did not significantly affect RT for trials in which participants had to use social cues (DP condition) to guide decisions (*p*=0.30; figure 3*a*). However, the three-way interaction revealed a cognitive load effect on RT when participants had to take into account a different perspective specifically when using social cues to select the correct object. In the DP condition, there was a significant cognitive load × perspective interaction (*p*<0.001), which was not observed in the DA condition (*p*=0.60) where cognitive load slowed RT across both perspectives. Following the significant interaction in DP showed that when under high cognitive load, participants were slower when taking a different perspective compared with when they did not have to take a different perspective (*p*<0.001). The best-fitting model did not include age group as a main or interactive effect for RT.

## 4. Discussion

In this study, we investigated how the availability of cognitive resources affects the ability to perform a social interaction task while simultaneously keeping track of non-social information—a form of multitasking—in adolescents and adults. Given the natural limitations of how much information individuals can keep track of at once, we hypothesized that multitasking performance in the social interaction task would be diminished when participants had to simultaneously keep track of several pieces of non-social information (high cognitive load). The results of the present study were in line with this hypothesis, as participants in both age groups were less proficient at performing both the social and non-social task when they were under high cognitive load. Compared with adults, the adolescent group showed a greater decrement in performance when having to keep track of several pieces of information while multitasking.

Our results suggest that processing and using social cues to guide behaviour is relatively automatic when the partner in a social interaction shares the same perspective (figure 3*a*). Both adult and adolescent participants were, on average, faster and more accurate when using social cues to guide decisions, compared to when using non-social cues to guide decisions. However, multitasking accuracy was impaired when participants had to inhibit their own egocentric perspective to select the correct object. While cognitive load did not differentially affect multitasking accuracy under different perspectives, it did affect how fast participants reacted in our social interaction task (DP condition). Participants in both age groups were slower at taking another person’s perspective, compared to their own, when under high cognitive load. This effect was specific to social cues, suggesting that taking another’s perspective is a cognitively taxing activity that can be disrupted when one is simultaneously keeping track of several pieces of non-social information.

A previous study using a live version of the Director Task found that adults under high cognitive load made more errors during trials that required inhibiting their egocentric perspective to select the correct item [7]. However, unlike the present study, this previous study did not include a control condition (the DA condition) that accounts for the general processing demands of inhibiting the prepotent response to select the distractor object. Therefore, the previous study was unable to address whether the depletion of cognitive resources interacted specifically with the social processing demands of the Director Task [7]. Although Lin *et al*. interpreted their results as providing evidence that cognitive resource availability affects the ability to use theory of mind during social interactions, without the control condition it is not possible to interpret the results as specifically relating to the cognitive resources needed to take into account the perspective of another individual. It could be that the presence of a distractor object in general would impair a participant’s performance. In this study, we tested the effects of cognitive load on processing social information by including a cognitive load manipulation and a control condition matched on the general processing demands of selecting a target among distracting stimuli (the DA condition). Thus, we were able to specifically measure the impact of social perspective taking, above and beyond inhibiting a prepotent response (i.e. egocentric perspective), on selecting the correct object. Our study showed that taking another person’s perspective when it differs from one’s own necessitates cognitive resources that are depleted under cognitive load, and that this effect is not simply due to the general cognitive control demands required when choosing a target object in the presence of a distractor object. However, it is important to note that this interaction was specific to RT, as the effect of cognitive load on accuracy in selecting the correct object was similar in both the DP and DA conditions.

We hypothesized that the natural variability between individuals in both cognitive resource capacity (WM capacity) and the tendency to take another’s perspective (TPT) would relate to performance on the multitasking paradigm. To test this hypothesis, we created a combined measure of these two traits and included it in our statistical model selection procedure. The best-fitting model included this combined trait, suggesting that trait differences were able to explain multitasking performance above and beyond other task-related factors. This is not surprising, given that our multitasking paradigm was a combination of two tasks that necessitated keeping track of non-social information and accurately taking another’s perspective. This was true for both adolescents and adults.

As adolescence is a time when complex social cognitive skills are changing along with the social environment (see [1] for review), this study sought to address how cognitive control abilities (i.e. the ability to manipulate information in WM) might influence the continued development of social navigation skills between adolescence and adulthood. An fMRI study that used a similar version of the Director Task found that adults recruited a network of fronto-parietal regions involved in cognitive control when inhibiting an egocentric perspective (across both the DP and DA conditions) more than did adolescents [12]. It might be that increased recruitment of cognitive control capacities during social interactions between adolescence and adulthood can offset the potential decrement in multitasking performance when individuals are placed under high cognitive load.

Within the typical social environment, we are regularly faced with situations that require multitasking while engaging in a social interaction. Therefore, it is important to understand how simultaneously keeping in mind extraneous information influences our ability to engage in a communicative task with another person. The results of this study support the idea that adolescents are more sensitive to additional cognitive load requirements than are adults in both social and non-social multitasking situations. These findings suggest that adolescents might have difficulty in certain social situations in which adults perform without any problem, such as when having to keep track of extraneous information while also socially interacting with another individual. Given that performance deficits resulting from multitasking interference effects occur only when an individual’s cognitive resources are sufficiently taxed [17], it might be that the high cognitive load condition in our study was more taxing for adolescents than for adults.

It is important to note that both adolescents and adults are affected by multitasking when engaging in social interactions that require perspective taking. Our results show that when participants had to keep track of only one piece of non-social information (low load), they were just as fast responding to the avatar’s directions regardless of the avatar’s perspective. However, when keeping track of three pieces of non-social information (high load), participants were significantly slower at responding to the avatar’s directions when the avatar did not share the same perspective as the participant. These results suggest that the natural pace of social interactions, such as everyday conversations, could be disrupted when adolescents or adults are simultaneously keeping track of extraneous information.

Our sample only included female participants in order to reduce the number of factors examined in this novel investigation. Future developmental studies investigating the effects of multitasking on performance should include both female and male participants in order to examine any potential sex differences.

In this study, participants kept track of either one or three pieces of non-social information while also performing a referential communication task with a computer avatar. This task is akin to having a conversation with someone while in the middle of an unrelated task—such as programming an experiment or doing a classroom assignment—in which one must keep track of information not relevant to the current social interaction. Our results show a significant decrease in accuracy in both social and non-social multitasking situations (approx. 10% for adults and approximately 15% for adolescents) when participants had to remember three pieces compared to one piece of non-social information. This suggests that attempting to keep track of just a few pieces of non-social information during a social situation can be impairing to both the social interaction and the later recall of the non-social information. Further, multitasking situations that some adults navigate effectively might be too difficult for some adolescents. These results might have implications for how adults who work with adolescents (e.g. teachers and mentors) structure activities with adolescents. For example, in-class group work might be particularly difficult for adolescents who are already struggling with the assignment topic.

## Data accessibility

These data and our analysis procedure are freely available for public use: http://dx.doi.org/10.6084/m9.figshare.1098780.
